# Beltrami-net: domain-independent deep D-bar learning for absolute imaging with electrical impedance tomography (a-EIT)

**DOI:** 10.1088/1361-6579/ab21b2

**Published:** 2019-07-23

**Authors:** S J Hamilton, A Hänninen, A Hauptmann, V Kolehmainen

**Affiliations:** 1 Department of Mathematics, Statistics, and Computer Science, Marquette University, Milwaukee, WI 53233, United States of America; 2 Department of Applied Physics, University of Eastern Finland, Kuopio, Finland; 3 Research Unit of Mathematical Sciences, University of Oulu, Oulu, Finland; 4 Department of Computer Science, University College London, London, United Kingdom; 5 Authors to whom any correspondence should be addressed.

**Keywords:** deep learning, electrical impedance tomography, D-Bar method, Beltrami equation, post-processing, conductivity

## Abstract

**Objective:**

To develop, and demonstrate the feasibility of, a novel image reconstruction method for absolute electrical impedance tomography (a-EIT) that pairs deep learning techniques with realtime robust D-bar methods and examine the influence of prior information on the reconstruction.

**Approach:**

A D-bar method is paired with a trained convolutional neural network (CNN) as a post-processing step. Training data is simulated for the network using no knowledge of the boundary shape by using an associated nonphysical Beltrami equation rather than simulating the traditional current and voltage data specific to a given domain. This allows the training data to be boundary shape independent. The method is tested on experimental data from two EIT systems (ACT4 and KIT4) with separate training sets of varying prior information.

**Main results:**

Post-processing the D-bar images with a CNN produces significant improvements in image quality measured by structural SIMilarity indices (SSIMs) as well as relative *ℓ*_2_ and *ℓ*_1_ image errors.

**Significance:**

This work demonstrates that more general networks can be trained without being specific about boundary shape, a key challenge in EIT image reconstruction. The work is promising for future studies involving databases of anatomical atlases.

## 1. Introduction

Electrical impedance tomography (EIT) probes a body with low-amplitude electrical currents applied on surface electrodes. The surface measurements can then be used as inputs to solve a mathematical inverse problem to recover the internal electrical properties (conductivity and permittivity) of the object. As EIT is a low-cost, noninvasive imaging modality with no ionizing radiation, it has several medical and industrial applications, see Cheney *et al* (1999) and Mueller and Siltanen (2012). The image recovery task in EIT, recovering the internal conductivity from the surface electrode measurements, is a severely ill-posed nonlinear inverse problem thus requiring carefully designed reconstruction algorithms capable of handling incorrectly known boundary shape, electrode locations, and noise in the measured EIT data. The ill-posedness of the inverse problem often results in images with low spatial resolution or severe image corruption due to modeling errors in a minimization task. The D-bar method (Nachman 1996, Knudsen *et al* 2009) has been shown to be robust to modeling errors and noise (Murphy and Mueller 2009, Hamilton *et al* 2018).

By viewing these low-resolution, real-time (Dodd and Mueller 2014), D-bar images as convolutions of the true images one can develop and train a convolutional neural network (CNN) to learn the blurring inherent in the D-bar reconstruction process on data of that type. This idea was introduced in Hamilton and Hauptmann (2018) and tested on experimental EIT data for absolute imaging in 2D. There, the training data for the network was simulated from the forward EIT model:

(1) $$\nabla \cdot \sigma (z)\nabla u(z)=0,\;z\in \Omega \subset {\mathbb{R}}^{2}\;\sigma \frac{\partial u}{\partial \nu }=g,\;z\in \partial \Omega$$

using the electrode continuum model (Hyvönen 2009, Hauptmann 2017) based on continuum current/voltage data computed from a known circular domain boundary. The trained network was then directly applied to D-bar reconstructions from the experimental data with no transfer training required. By contrast, here we simulate our training data from the associated, non-physical, Beltrami problem (Astala and Päivärinta 2006a, 2006b) and ‘Shortcut D-bar Method’ (Astala *et al* 2014) to remove any knowledge of the boundary (shape and electrodes) from the training process. We test the network on EIT data from two different EIT machines (ACT4 (Liu *et al* 2005) and KIT4 (Kourunen *et al* 2008)) with different boundary shapes. In practice, a network could be constructed using a database of CT scans where all that is needed is approximate internal structure boundaries (heart, lungs, spine, etc) and reasonable conductivity value windows for each type of inclusion. The CTs could be scaled such that the maximum radial component of the thorax boundary is one. Alternatively, one could bypass any direct incorporation of organs by instead training using inclusions of ellipses, circles, etc. The patient-specific voltage and current EIT data would then be scaled to correspond to a maximum radius of 1 by scaling the associated DN (or ND) matrix by the largest radial component of the patient’s approximated boundary shape (see Isaacson *et al* (2004)). In this study we investigate the particular question of how informative the training data needs to be in order to perform the desired image enhancement task after an initial reconstruction. That means, we consider two different scenarios in this study.

- Thoracic measurements for a human patient, here a database can be built from anatomical atlases. In this setting the imaging task is highly constrained by anatomical features and hence training data can be tuned to be specific for this particular task. This constitutes a case of high *a priori* knowledge. We consider tank data with thoracic specific agar targets.
- Assessment of more generic training data without any anatomical prior information, with which we are able to achieve sufficient reconstruction quality for a vast application area. This can be considered a more generic task with low level of *a priori* information.

Due to the ill-posedness and non-linearity of the EIT problem, the resolution and practical utility of the EIT images is basically dependent on the amount of prior information available and how well one is able to transform the prior information and related uncertainties into a computationally useful form. The literature contains a number of approaches for utilizing prior information, including regularization-based techniques (Vauhkonen *et al* 1998, Kaipio *et al* 1999, Borsic *et al* 2002, Kolehmainen *et al* 2019), Bayesian approaches (Kaipio *et al* 2000) as well as prior informed D-bar methods (Alsaker and Mueller 2016, Alsaker *et al* 2018), which all produce high quality solutions and have different technical benefits and intricacies. For example, considering a case where one would have prior information available in form of a set of plausible sample images from an anatomical atlas, the problem in the Bayesian setup would be how to transform the set of sample images into a form of a prior density model. The purpose of the present study is to propose a new kind of approach for an accurate EIT reconstruction. The key ingredient of the proposed approach is to train a CNN for post-processing enhancement of a standard EIT reconstruction (which has poor resolution). One feature of the proposed approach is that it allows straightforward inclusion of sample-based prior information into the learning process. This can be particularly advantageous in the cases where the prior is available only in form of a set of plausible solutions, such as set of images from an anatomical atlas, instead of having a parametric model for the prior density. The proposed approach allows straightforward utilization of the samples as input to the learning process.

The application of deep learning methods, in particular convolutional neural networks (CNNs), has attracted major attention in recent years and shows great promise for improving images in tomographic reconstruction tasks. The most prominent approach, which we follow here as well, is given by post-processing of an initial reconstruction based on an analytic inversion formula, such as filtered back-projection in x-ray CT (Jin *et al* 2017, Kang *et al* 2017). Other promising clinical applications of this approach are dynamic cardiovascular magnetic resonance imaging (Schlemper *et al* 2018, Hauptmann *et al* 2019). Recent studies, in addition to Hamilton and Hauptmann (2018), have explored the possibility of using deep learning for EIT with artificial neural networks (Martin and Choi 2017) and variational autoencoders for lung imaging (Seo *et al* 2018). Furthermore, several studies propose combining iterative variational techniques with deep learning to obtain superior reconstruction quality and more flexible generalization by including the forward operator in the network architectures (Adler and Öktem 2017, Hammernik *et al* 2018, Hauptmann *et al* 2018). In this study we follow the approach discussed in Hamilton and Hauptmann (2018), but without the need for boundary shapes in the training data. We proceed to compare our results to variational techniques with comparable amount of prior information for both imaging scenarios mentioned above.

Section 2 presents the methods used in this work including the proposed new algorithm and how reconstruction quality will be assessed. Results of the proposed method on experimental EIT tank data from ACT4 and KIT4 are presented in section 3 and conclusions drawn in section 4.

## 2. Methods

Here we consider the 2D real-valued conductivity EIT problem

(2) $$\nabla \cdot \sigma (z)\nabla u(z)=0,\;z\in \Omega \subset {\mathbb{R}}^{2},$$

where *σ* = *σ*(*z*) is the spatially dependent conductivity and *u* = *u*(*z*) the electric potential. The current and voltage measurements take the form of approximate knowledge of the Neumann-to-Dirichlet (ND) map $R_{\sigma }:\sigma \frac{\partial u}{\partial \nu }\mapsto g$ for *z* ∈ *∂*Ω which maps a boundary current to the corresponding boundary voltage, and *ν* = *ν*(*z*) denotes the outward unit normal vector to *∂*Ω. Here, for simplicity, we assume the conductivity is constant *σ* = *σ*_0_ in a neighborhood of the boundary. If *σ* is not constant near *∂*Ω, a padding of the domain can be used as in Nachman (1996), Siltanen and Tamminen (2016) reducing the problem back to the case studied here.

The ND map $R_{\sigma }$ can be approximated from the measured current and voltage data with the matrix *R*_*σ*_:

(3) $$R_{\sigma }(m,n):=\sum_{l=1}^{L}\frac{\phi_{l}^{m}v_{l}^{n}}{\left|e_{l}\right|},\;1⩽m,n⩽{\text{num}}_{\text{LI}},$$

where *L* denotes the number of electrodes used, *num*_*LI*_ is the number of linearly independent current patterns applied (maximum is *L* − 1), and *ϕ*^*m*^, and *v*^*n*^ denote the normalized *m*th current pattern vector and *n*th voltage vectors (see Isaacson *et al* (2004) and Hamilton *et al* (2018) for scaling details). The methods described below assume the boundary conductivity *σ*_0_ = 1 and that the domain has a maximum radial component of 1. However, if this is not the case for the measured data, the ND matrix *R*_*σ*_ can be scaled appropriately, as described in Isaacson *et al* (2004), reducing the problem to the case studied here.

### 2.1. Intro to D-bar methods for 2D EIT

While various D-bar-based reconstruction algorithms for 2D EIT exist, they all have the same main structure:

$$[\text{CURRENT }\&\text{ VOLTAGE DATA}]\;\overset{1}{\to }\;[\text{SCATTERING DATA}]\;\overset{2}{\to }\;[\text{CONDUCTIVITY}].$$

The scattering data is non-physical, and can be thought of as a nonlinear Fourier transform. The D-bar methods differ in the particular formulas used to compute the scattering data and recover the conductivity. D-bar methods come from inverse-scattering theory, an area of mathematics that brought the elegant solution to the Korteweg–de Vries (KdV) equation. D-bar methods for EIT get their name from a $\bar{\partial }$ (D-bar) equation used to recover the conductivity *σ* in Step 2 above.

Here we simulate our training data using using a variation of the ‘shortcut D-bar method’ (Astala *et al* 2014) which blends the D-bar method from the Schrödinger equation and that of the Beltrami equation. This is done to allow us to train the network using *L*^∞^ conductivities (Beltrami method) but still reconstruct the conductivity from the scattering data using the Schrödinger ${\bar{\partial }}_{k}$ equation which (Astala *et al* 2014) suggest is more robust than Step 2 of the Beltrami method. A recent paper by Lytle *et al* (2018) in fact prove that the integral equations in the Schrödinger formulation of the D-bar method hold for *L*^∞^ conductivities which are one near *∂*Ω.

#### 2.1.1. Algorithm for simulating the training data

Let Ω be the unit disc. Given a set of *N* conductivities ${\left\{\sigma_{n}\right\}}_{n=1}^{N}$ in *L*^∞^(Ω), for each *σ*_*n*_ compute the associated low-pass D-bar reconstruction $\sigma_{n}^{\text{DB}}$ as follows: (1) generate the Beltrami scattering data *τ*(*k*) for |*k*| ⩽ *R* for some chosen radius *R* > 0, and (2) solve the Schrödinger ${\bar{\partial }}_{k}$ equation using the Beltrami scattering data for |*k*| ⩽ *r* where *r* ⩽ *R*.

*Step 1*: Generate the Beltrami scattering data *τ*_*n*_(*k*) for *σ*_*n*_(*z*) for k∈ℂ, |*k*| ⩽ *R* as in Astala *et al* (2014)

(4) $$\bar{\tau_{n}(k)}:=\frac{1}{2\pi }\int_{{\mathbb{R}}^{2}}{\bar{\partial }}_{z}[M_{+\mu_{n}}(z,k)-M_{-\mu_{n}}(z,k)]dz_{1}dz_{2},$$

where $M_{\pm \mu_{n}}(z,k)=e^{-ikz}f_{\pm \mu_{n}}(z,k)$ are solutions to the Beltrami equation

(5) $${\bar{\partial }}_{z}f_{\pm \mu_{n}}(z,k)=\pm \mu_{n}(z)\bar{\partial_{z}f_{\pm \mu_{n}}(z,k)}$$

satisfying $M_{\pm \mu_{n}}(z,k)=1+O\left(\frac{1}{\left|z\right|}\right)$ for large |*z*| and $\mu_{n}(z)=\frac{1-\sigma_{n}(z)}{1+\sigma_{n}(z)}$ denotes the corresponding Beltrami coefficient. Note that $-\mu_{n}(z)=\frac{1-\frac{1}{\sigma_{n}(z)}}{1+\frac{1}{\sigma_{n}(z)}}$ as in Astala *et al* (2014).

*Step 2*: Relate the Beltrami and Schrödinger scattering data via $t_{n}(k)=-4\pi i\bar{k}\tau_{n}(k)$, setting **t**_*n*_(*k*) = 0 for all |*k*| > *R*. Recover the low-pass D-bar reconstruction $\sigma_{n}^{\text{DB}}={\left[m_{n}(z,0)\right]}^{2}$ by solving the Schrödinger ${\bar{\partial }}_{k}$ equation (Knudsen *et al* 2009)

(6) $${\bar{\partial }}_{k}m_{n}(z,k)=\frac{1}{4\pi \bar{k}}t_{n}(k)e(z,-k)\bar{m_{n}(z,k)},$$

for each *z* ∈ [−1,1]^2^, where $e(z,k):=\text{exp}\{i(kz+\bar{k}\bar{z})\}$ is a unitary multiplier, using the integral form

(7) $$m_{n}(z,\kappa )=1+\frac{1}{4\pi^{2}}\int_{\mathbb{C}}\frac{t_{n}(k)e(z,-k)}{(\kappa -k)\bar{k}}\bar{m_{n}(z,k)}d\kappa_{1}d\kappa_{2},$$

and the computational method outlined in Mueller *et al* (2002) and Astala *et al* (2014).

Note that no electrode or boundary information is used in the training data as *μ*_*n*_(*z*) = 0 near *∂*Ω. The choice of Ω=D does not include boundary specific information since in the reconstruction step from experimental data, we will scale the ND map by the maximum radial component of the experimental domain Ω_meas_, shrinking the problem to exist within our studied domain Ω=D. Additionally, note that the integral in (7) reduces to an integral over |*k*| ⩽ *R* due to the compact support of **t**_*n*_(*k*), and from Nachman (1996) $\frac{{\text{t}}_{n}(k)}{k}=0$ for *k* = 0.

#### 2.1.2. Recovery of conductivity from experimental data

Recover the D-bar reconstruction *σ*^DB^ from the measured current and voltage data via a modification to the Schrödinger **t** ‘exp’ method as follows.

*Step 1*: Compute the modified Schrödinger ‘exp’ scattering data

(8) $$t^{\text{exp}}(k)=\int_{\partial \Omega_{1}}e^{i\bar{k}\bar{z}}\left(\Lambda_{\sigma }-\Lambda_{1}\right)e^{ikz}ds(z)\;=\int_{\partial \Omega_{1}}e^{i\bar{k}\bar{z}}\left[\Lambda_{\sigma }\left(e^{ikz}\right)-ik\nu e^{ikz}\right]ds(z),$$

for k∈ℂ\0, |*k*| ⩽ *R*_meas_ for some chosen radius 0 < *R*_meas_ ⩽ *R*.

*Step 2*: Recover the D-bar conductivity reconstruction *σ*^DB^ = (*m*^exp^(*z*,0))^2^ using (6) with **t**^**exp**^ in place of **t**_*n*_, setting $\frac{{\text{t}}^{\text{exp}}(k)}{\bar{k}}=0$ for *k* = 0.

The second line (8) comes from computing Λ_1_*e*^*ikz*^ = 1∇ (*e*^*ikz*^) · *v* = *ikve*^*ikz*^ which uses a continuum approximation for the DN map Λ_1_ where *ν* = *ν*(*z*) is the unit outward facing normal to the scaled boundary *∂*Ω_1_ which has maximal radial component 1. The DN matrix approximation to Λ_*σ*_ is computed from *L*_*σ*_ = (*R*_*σ*_) ^−1^ via (3). The DN map is also scaled by the radius of the smallest circle containing the imaged domain Ω_meas_, and *σ*_0_ the conductivity near the boundary *∂*Ω_meas_. If *σ*_0_ is unknown, the best constant-conductivity fit to the measured data can be used as described in Cheney *et al* (1990). The resulting conductivity at the end of the algorithm is then re-scaled by *σ*_0_. Here we compute *ν* numerically using a parameterization of the approximate boundary shape function (see Hamilton *et al* (2018) for robustness studies of D-bar methods to incorrect boundary shape). Note that we only require the measured current and voltage data, approximate boundary shape of the imaged domain Ω_meas_, and approximate locations of the electrodes for the D-bar reconstruction *σ*^DB^.

#### 2.1.3. Why choose the Beltrami approach?

Inspired by the success of the ‘Deep D-bar’ approach in Hamilton and Hauptmann (2018), we chose to again use a low-pass D-bar image as as starting point due to their real-time capabilities and general blurry but reliable reconstructions. By training a CNN with data/reconstructions from the Beltrami equation (5) rather than by using a FEM approach on the traditional conductivity equation (2), the trained CNN does not dependent on a specified domain boundary making the approach more general and theoretically reducing the need to re-train the network for individuals of different domain shapes. This is due to the fact that the conductivity is assigned to a constant value outside of the organs. Since $\mu (z)=\frac{1-\sigma }{1+\sigma }$, and $-\mu (z)=\frac{1-\frac{1}{\sigma (z)}}{1+\frac{1}{\sigma (z)}}$, and we scale *σ* such that it has a background value of 1 in the Beltrami problem, this makes *μ* = 0 outside the organs and removes the issue of the domain boundary completely from the problem. This has the advantage of, e.g. in thoracic imaging, being able to use a more generally trained CNN from an anatomical atlas that does not require the patient to have the same domain boundary as what was used to train a FEM-based network. Alternative approaches could of course be used where the FEM-based reconstructions are created from various domain boundaries as well, however this may increase the size of the training data and is outside the scope of this study.

### 2.2. Deep learning and image reconstruction

The driving motivation to use deep learning methods in imaging and in particular for image reconstruction is motivated by the limitation of hand-crafted priors in variational and statistical reconstruction methods. By training a network on data that represents the desired images, we can learn more general data-driven representations, also referred to as the learned data manifold. The draw back of learning-based methods is, clearly, that these learned priors are only implicit and do not have an analytical representation.

Applications in tomographic image reconstruction can be roughly divided into three categories.

- Fully learned: a mapping from data to reconstruction is learned without the need of a model (after training).
- Model enforced: direct reconstruction by an analytically known and understood reconstruction procedure, ideally a regularization strategy, followed by learned post-processing.
- Model-based: reconstruction in a cascaded sense, where the model information is used repeatedly. Typically these are given as learned iterative reconstruction algorithms.

Even though fully learned reconstruction methods have been studied and demonstrate promising results (Martin and Choi 2016, Zhu *et al* 2018), this approach neglects any model knowledge and hence analytically known robustness results. In contrast, using the model in approach (b) and (c) retains known properties and stability results. Additionally, for EIT it was shown to improve stability (Martin and Choi 2017), especially for reconstructions from measurement data. In this study we chose to use the D-bar algorithm, a known regularization strategy for EIT (Knudsen *et al* 2009), as starting point to have stability in the input to the network. For the network architecture we chose the very successful U-net architecture (Ronneberger *et al* 2015), a multiscale convolutional neural network. This particular network architecture has been proposed by Kang *et al* (2017) and Jin *et al* (2017) for post-processing corrupted reconstructions, and has been shown to be successful in the application to a variety of tomographic problems Antholzer *et al* (2018) and Hauptmann *et al* (2019), but has also been the focus of analytical studies Ye *et al* (2018). Thus, we follow the incentive to combine a robust regularization strategy with a well established, and partially understood, network architecture for reconstruction in our application.

#### 2.2.1. Beltrami-net for absolute EIT

In this study we follow the approach of post-processing corrupted reconstructions, which in our case are given by the D-bar algorithm described above in section 2.1. This methodology is motivated by the fact that the initial reconstruction is of convolutional type, such as the normal operator in CT, or in our case inversion of the truncated scattering transform, that can be interpreted as nonlinear Fourier transform. Consequently, we follow (Jin *et al* 2017) where the authors propose that a CNN can be used to remove artefacts and recover resolution loss present in the initial reconstruction.

Let us denote the used U-net architecture by *G*_Θ_, where Θ are the learnable network parameters consisting of convolutional filters and biases, see Goodfellow *et al* (2016) for an introduction. Then the supervised learning task is given as the optimization problem to find an optimal set of parameters, such that a loss function is minimized with respect to the training set. Specifically, in our case the training set is given by ground truth conductivities *σ*_*n*_ and corresponding D-bar reconstructions $\sigma_{n}^{\text{DB}}$ for n∈N={1,…,N}, both given on the square [−1,1]^2^. We remind that the D-bar reconstructions for this training set are obtained from the Beltrami scattering data as outlined in section 2.1.1. Given this training set, the aim is to find network parameters, such that *G*_Θ_ maps from D-bar reconstructions to the correct ground truth conductivity. Thus, we aim to find an optimal set of parameters as

(9) $$\Theta =\text{arg}\underset{\Theta }{\text{min}}\sum_{n=1}^{N}{\left\|G_{\Theta }\left(\sigma_{n}^{\text{DB}}\right)-\sigma_{n}\right\|}_{2}^{2}.$$

The optimization is typically performed in subsets (batches) of training pairs ${\left\{\sigma_{n},\sigma_{n}^{\text{DB}}\right\}}_{I\subset N}$, rather than the whole training set. Details on the specific training data and the training procedures are given in section 2.4.

The chosen network architectures differ slightly depending on which task, (i) or (ii), of the section 1 is considered. For scenario (i) the thoracic imaging task, we employ the same network architecture as described in Hamilton and Hauptmann (2018) as it has been shown to be specifically suited to reproduce structures in a known constrained environment with strong prior information. For task (ii) with minimal *a priori* knowledge, an assessment of network architectures was performed and we found that adding a residual connection as in Jin *et al* (2017) increased robustness in recovering more general shapes that were not present in the training set. In both cases we kept the filter size of the convolutional kernels as 5 × 5 and used four max-pool layers, as the original U-Net architecture suggests. Networks are implemented with TensorFlow in Python^6^.

### 2.3. Evaluation of the method

To evaluate the effectiveness of our proposed Beltrami-net method we tested it on experimental data from two different EIT machines, namely, ACT4 from Rensselaer Polytechnic Institute (RPI) (Liu *et al* 2005) and KIT4 from the University of Eastern Finland (UEF) (Kourunen *et al* 2008). We evaluate reconstruction quality using *structural SIMilarity* Indices (SSIMs) and relative *ℓ*_1_ and ℓ_2_ image errors. The ground truth inclusion boundaries were extracted from photographs of the experiments. We compare the Beltrami-net reconstructions to the classical low-pass D-bar reconstructions as well as (structured) total variation reconstructions.

#### 2.3.1. Comparison to variational methods

To compare the results to regularization-based absolute EIT reconstructions, we include 2D reconstructions using a regularized non-linear least squares formulation

(10) $$\hat{\sigma }=\text{arg}\underset{\sigma >0}{\text{min}}\left\{\left\|V-U(\sigma ){\|}^{2}+\alpha \Psi (\sigma \right)\right\},$$

where Ψ(*σ*) is a *structured total variation* (STV) regularization functional (Kolehmainen *et al* 2019), defined as

(11) $$\Psi (\sigma )=\int_{\Omega }\sqrt{\|\nabla \sigma {\|}_{B(p)}^{2}+\beta }\text{d}r,$$

where *p*(*r*) is an auxiliary reference image and *B*(*p*) is a symmetric matrix valued mapping which is used to incorporate prior information from the reference image and *β* is a smoothing parameter. In a nutshell, the idea is to choose the mapping *B*(*p*) such that the regularization promotes similar alignment of structures (represented by the level sets) of the unknown *σ* and the reference image *p*. Following (Kolehmainen *et al* 2019), we define

(12) $$B(r)=I-(1-\gamma (r))\nu (r)\nu {\left(r\right)}^{\text{T}},$$

where

(13) $$\nu (r)=\{\begin{array}{ll} 0 & \text{ if }\|\nabla p(r)\|\;=\;0\\ \nabla p(r)/\|\nabla p(r)\| & \text{ otherwise } \end{array}$$

is a vector field (normal to the level sets of *p*) and

(14) $$\gamma (r)=\{\begin{array}{ll} 0.025 & \text{ when }\|\nabla p(r)\|\;>\;0\\ 1 & \text{ otherwise } \end{array}$$

is an edge weighting function which is designed to promote a small penalty for changes in *σ* in locations where *p* exhibits changes. The discretization of the method (10) is based on the finite element method (FEM) and the non-linear optimization is solved by a lagged Gauss–Newton method equipped with a line search algorithm. The line search is implemented using bounded minimization such that the non-negativity *σ* > 0 is enforced. The regularization parameter *α* was tuned manually for the best visual quality of the reconstruction. For more details of the method, see Kolehmainen *et al* (2019).

#### 2.3.2. Experimental data

**Figure 1.**
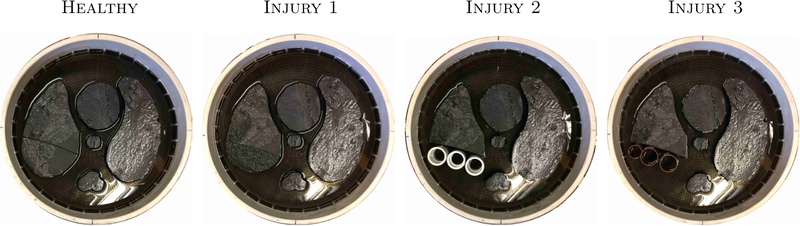
The experimental setups for the ACT4 data collection. Four scenarios were tested beginning with a ‘Healthy’ setup: conductive heart and aorta, resistive lungs and spine. In ‘Injury 1’, the bottom portion of the right (DICOM orientation) lung was removed and replaced with a conductive agar target matching the conductivity of the heart/aorta. In ‘Injury 2’, the removed portion of the right lung was replaced with three plastic pipes and for ‘Injury 3’ the removed portion is replaced with three copper pipes.

Archival ACT4 data, taken on a circular tank of radius 15 cm with 32 electrodes (width 2.5 cm), was used. Agar targets with added graphite were placed in a saline bath (0.3 S m^−1^) filled to a height of 2.25 cm. Conductive and resistive targets were used to simulate the heart and aorta, as well as the lung and spine, respectively. See figure 1 for the experimental setups. Table 1 displays the measured conductivities of the targets, using test-cells, computed via Impedimed’s SFB-7 bioimpedance meter^7^. Trigonometric voltage patterns, with maximum amplitude 0.5 V, were applied at a frequency of 3 kHz and the resulting currents measured. For consistency with previous studies, a change of basis was performed on the measured current and voltage data to synthesize the data that would have occurred if current had been applied instead of voltage (see Hamilton and Hauptmann (2018)). The ND and DN matrices were then computed as described in section 2, equation (3).

**Figure 2.**
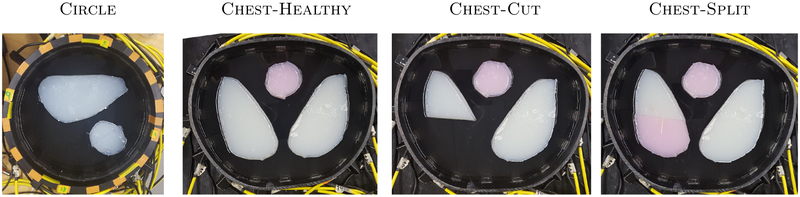
Experimental setups for the KIT4 data on three different experimental tank setups. Circle: The large object is low conductivity and small object is high conductivity. Chest: The agar targets are either high (pink) or low (white) conductivity.

We collected KIT4 data using two different, translationally symmetric tanks to obtain data for two different boundary shapes, namely circle and chest-shaped, as shown in figure 2. In each tank, the number of electrodes is sixteen. Adjacent (skip-0) current patterns were applied with current frequency at 10 kHz and amplitude 3mA. Conductive and resistive agar targets were used across all the KIT4 experiments. The circular tank has a radius of 14 cm with 16 electrodes of width 2.5 cm. Agar targets of conductivity 67 mS m^−1^ (large object on the top) and 305 mS m^−1^ (smaller, nearly circular object on the bottom right) were placed in a saline bath of conductivity 135 mS m^−1^ filled to a height of 45 mm. The chest shaped tank has a perimeter of 1.02m with 16 electrodes of width 2 cm attached. The locations of the electrodes are not exactly equidistant from one another but can be seen from the photographs (see figure 2). Agar targets consisting of high conductivity 323 mS m^−1^ (targets with pink ink) and low conductivity 61 mS m^−1^ (white) were placed in a saline bath (conductivity 135 mS m^−1^, height 47 mm for the *chest-healthy* and *chest-cut* targets, and 44 mm for the *chest-split* target in figure 2). The right (DICOM) lung was cut and two simulated injuries explored: (1) the bottom portion was removed completely (figure 2: *chest-cut*) and (2) the bottom portion was replaced with a higher conductivity piece of agar (figure 2: *chest-split*).

### 2.4. Training data

Two sets of training data were used in this study, tailored to the ACT4 and KIT4 experiments. We introduce the notation $\tilde{\sigma }$ to denote a conductivity that has not yet been scaled to a boundary conductivity of 1, reserving *σ* solely for conductivities with a boundary value of 1.

#### 2.4.1 ACT4 phantoms

**Figure 3.**
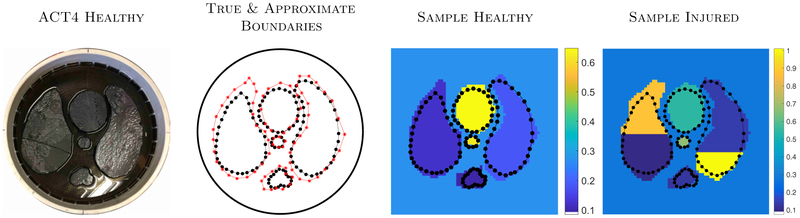
Samples of the simulated conductivities used to generate the ACT4 training data corresponding to the experiments shown in figure 1. Starting with a healthy setup (left), the ‘true organ boundaries’ (shown in black dots) were extracted from the photograph along with an ‘approximate organ boundaries’ (red stars) which are displayed in the second image. Noise was added to these approximate boundary points to generate the organ boundaries used in the simulated conductivities. Samples of such conductivities are shown in the third and fourth images with the true organ boundaries outlined in black dots.

Candidate phantoms ${\tilde{\sigma }}_{n}$ for the ACT4 training were formed by extracting the approximate boundaries of the inclusions from the ‘Healthy ‘ setup shown in figure 3 (first). The approximate boundaries are shown in red * and the true boundaries are shown in black dots (figure 3, second). Phantoms ${\tilde{\sigma }}_{n}$ were generated as follows.

- *Determine which objects are included*. Random numbers were generated from the uniform distribution on [0,1] to determine whether each inclusion (left lung: 90%, right lung: 90%, spine: 100%, heart: 95%, aorta: 95%) was included in ${\tilde{\sigma }}_{n}$.
- *Determine the conductivities of each target in* ${\tilde{\sigma }}_{n}$. The conductivities were assigned by drawing random numbers from uniform distributions using the respective conductivity windows outlined in table 1.
- *Determine the locations of each target in* ${\tilde{\sigma }}_{n}$. The coordinates of the each inclusion were created by adding noise, using the awgn command in Matlab, to the ‘approximate’ coordinates (red stars) of the corresponding inclusion, see figure 3.

As the ACT4 experiments contained ‘injuries’ to the right (DICOM) lung, simple injuries were simulated in the training data as follows. For each included lung, do the following.

- *Determine if the given lung contains an injury*. Generate a random number to determine whether or not an injury took place in the lung (50% chance).
- *If yes, divide the lung into two regions*.. Create a horizontal dividing line randomly by using the max and min vertical *x*_2_ coordinates of the lung dividing the lung into two regions.
- *Assign the injury*. Draw a random number to determine which region (top or bottom) the ‘injury’ took place (50–50 chance), and another random number drawn from the uniform distribution on the interval [0.01,1.5] to determine the conductivity of the injured region.

**Figure 4.**
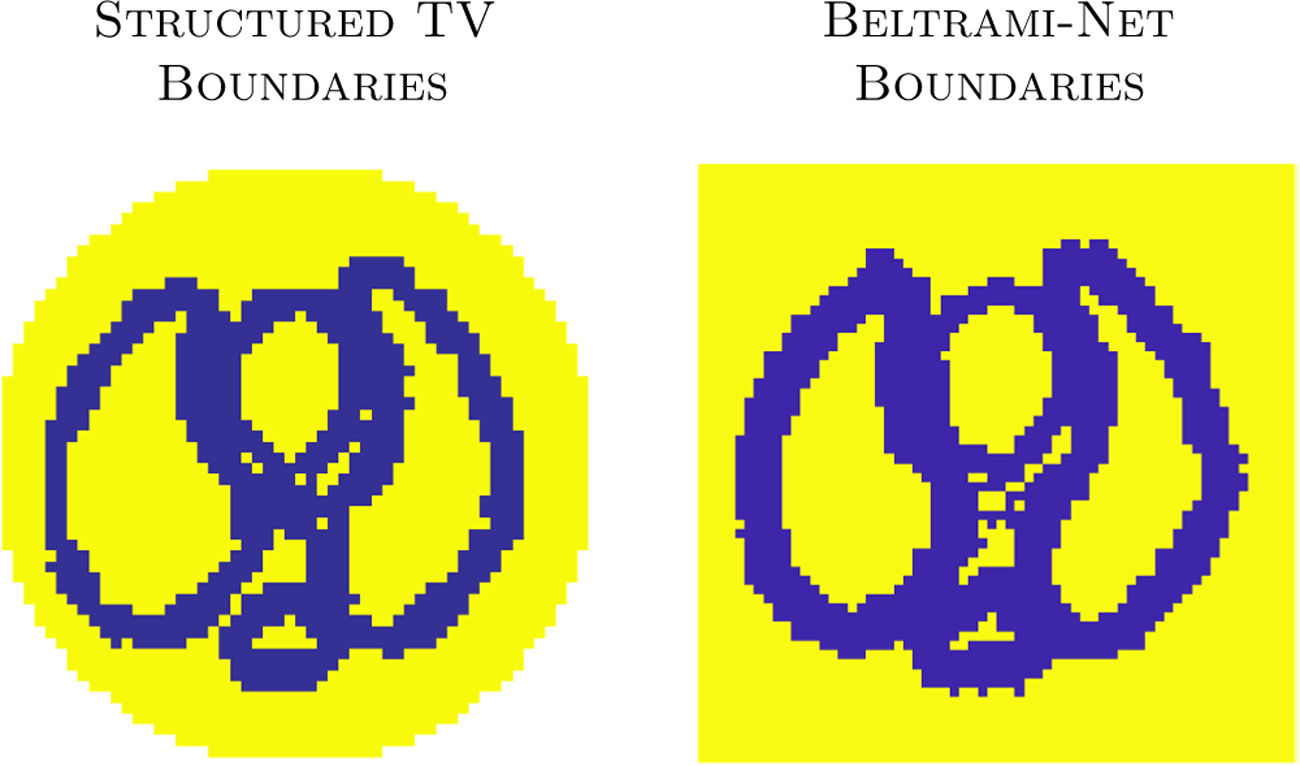
Comparison of Structured TV (STV) prior organ boundaries and boundaries extracted from Beltrami-net training data. Note this excludes the ‘cuts’ simulated for the training data of Beltrami-net. The image on the left is the weighting function *γ*(*r*) for the STV, equation (14)).

More complicated injuries were not considered here to allow for direct comparison to the previous study (Hamilton and Hauptmann 2018). Sample phantoms *σ*_*n*_ can be seen in figure 3, third and fourth images. The range in which organ boundaries are sampled for the training data is illustrated in figure 4, not including ‘cuts’. Additionally we show weighting function used for the structured TV reconstructions, representing a smiliar amount of priort information on where organ boundaries are expected.

#### 2.4.2. KIT4 phantoms

**Figure 5.**
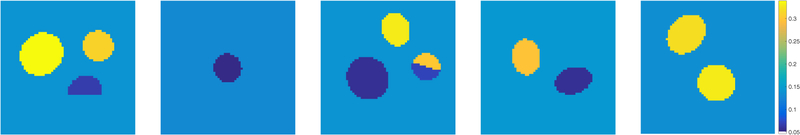
Samples of the simulated conductivities used to generate the KIT4 training data corresponding to the experiments shown in figure 2. One to three ellipses of varying eccentricities were randomly included with the possibility of inclusions being divided into two pieces of with no portion smaller than 1/4 of the original inclusion.

Conductivity phantoms ${\tilde{\sigma }}_{n}$ for the KIT4 training data were more general as the sizes and locations of the targets in the experiments varied greatly. Phantoms consisted of one to three ellipses of varying size (semi-major and minor axes chosen from the uniform distribution on [0.2,0.35]), location *ρe*^*iθ*^ for *ρ* ∈ [0,0.6] and *θ* ∈ [0,2*π*), and angular orientation in [0,2*π*). The ellipses were not permitted to overlap, and were all forced to be completely contained inside a *z*-disc of radius 0.95. The background conductivity was chosen from the uniform distribution on the interval [0.13,0.145]. For each inclusion, a random number was drawn to determine whether the inclusion was more or less conductive than the background (50–50 chance) and conductivities randomly assigned from the corresponding uniform distributions [0.29,0.34] and [0.05,0.075]. The chance of a target being split into two pieces was 1 in 3. If split, no region could be smaller than 1/4 the size of the whole inclusion, and the split could be along any dividing line (horizontal, diagonal, vertical). Divided inclusions were forced to either (1) have one part match the conductivity of the background, or (2) be split into a portion that is more conductive than the background and a portion that is less conductive than the background. Sample simulated conductivities ${\tilde{\sigma }}_{n}$ are shown in figure 5.

#### 2.4.3. Producing training data

For each conductivity phantom ${\tilde{\sigma }}_{n}$, the conductivity was scaled to a boundary value of 1 via $\sigma_{n}=\frac{1}{\sigma_{b_{n}}}{\tilde{\sigma }}_{n}$ where $\sigma_{b_{n}}$ denotes the constant conductivity near the the boundary, here the constant background value. If using a more complicated anatomical atlas, the value for $\sigma_{b_{n}}$ would be the constant conductivity for the tissue at the patient’s boundary. Then, the conductivity is extended to [−1,1]^2^ by setting *σ*_*n*_ = 1 for *z* ∈ [−1,1]^2^ \ Ω_*n*_. Then, for each scaled conductivity *σ*_*n*_, the Beltrami scattering data *τ*_*n*_(*k*) (4) was computed for |*k*| ⩽ *R*_ACT4_ = 5 or |*k*| ⩽ *R*_KIT4_ = 5.5, using a 2^5^ × 2^5^ uniformly spaced *k* − grid on [−5,5]^2^ or [−5.5,5.5]^2^, respectively, by solving (5) with Beltrami coefficients $\mu_{n}(z)=\frac{1-\sigma_{n}(z)}{1+\sigma_{n}(z)}$ and $-\mu_{n}(z)=\frac{1-\frac{1}{\sigma_{n}(z)}}{1+\frac{1}{\sigma_{n}(z)}}$ as outlined in step 1 of section 2.1.1. Next, the blurred D-bar reconstruction $\sigma_{n}^{DB}$ was recovered by step 2 of section 2.1.1 as follows. First, the Beltrami *τ*_*n*_ was related to the Schrödinger **t**_*n*_ scattering data by $t_{n}(k)=-4\pi i\bar{k}\tau_{n}(k)$. Then, a random number *R*_*n*_ was generated for the new scattering radius cutoff from the uniform distribution on [3.5,5] for ACT4, or [4,5.5] for KIT4. Then, the computed scattering data **t**_*n*_ was interpolated to a new 2^6^ × 2^6^ *k* − *grid* with maximum radius *R*_*n*_ on [−*R*_*n*_, *R*_*n*_]^2^. A non-uniform cutoff threshold was enforced by setting **t**_*n*_(*k*) = 0 if |*Re*(**t**_*n*_(*k*)| or |*Im*(**t**_*n*_(*k*)| exceeded *thresh* = 24 or |*k*| > *R*_*n*_. Then, the ${\bar{\partial }}_{k}$ equation was solved using the integral form (7) and the D-bar conductivity recovered as $\sigma_{n}^{\text{DB}}(z)=\sigma_{b_{n}}{\left(m_{n}(z,0)\right)}^{2}$, rescaling by the boundary conductivity $\sigma_{b_{n}}$, using a 2^6^ × 2^6^ *z* − grid on [−1,1]^2^ with gridsize *h*_*z*_ ≈ 0.0317.

#### 2.4.4. Training the networks

A total of 4096 (ACT4) and 15360 (KIT4) pairs $\left\{{\tilde{\sigma }}_{n},\sigma_{n}^{\text{DB}}\right\}$ were created for use as training data in the U-net architectures described above in section 2.2. Training was performed with the Adam optimizer and an initial learning rate of 10^−4^ to minimize the *ℓ*^2^-loss (9) with a batch size of 16 and for a total of 200 000 iterations. Training was monitored with a simulated validation set of ~5% of the training set size. The long training time, in terms of iterations, was mainly necessary to obtain constant areas in the inclusions as well as background. The training procedure took roughly three hours for each experiment on a single Nvidia Titan XP GPU.

Then, after the successful training procedure, the effectiveness was evaluated on simulated datasets $\sigma_{n}^{\text{DB}}$ not used in the training or validation data (section 3.1) as well as experimental reconstructions for the ACT4 and KIT4 data, applied to the respective ACT4 or KIT4 network (section 3.2).

## 3. Results and discussion

Here we present the results of the new Beltrami-net method on experimental, as well as simulated, data from the ACT4 and KIT4 EIT systems.

### 3.1. Reconstructions from simulated data

We begin by visually testing the quality of the Beltrami-net approach on simulated data. We explore test cases consistent with the training data, as well as phantoms that deviate from the procedure for creating the training set.

**Figure 6.**
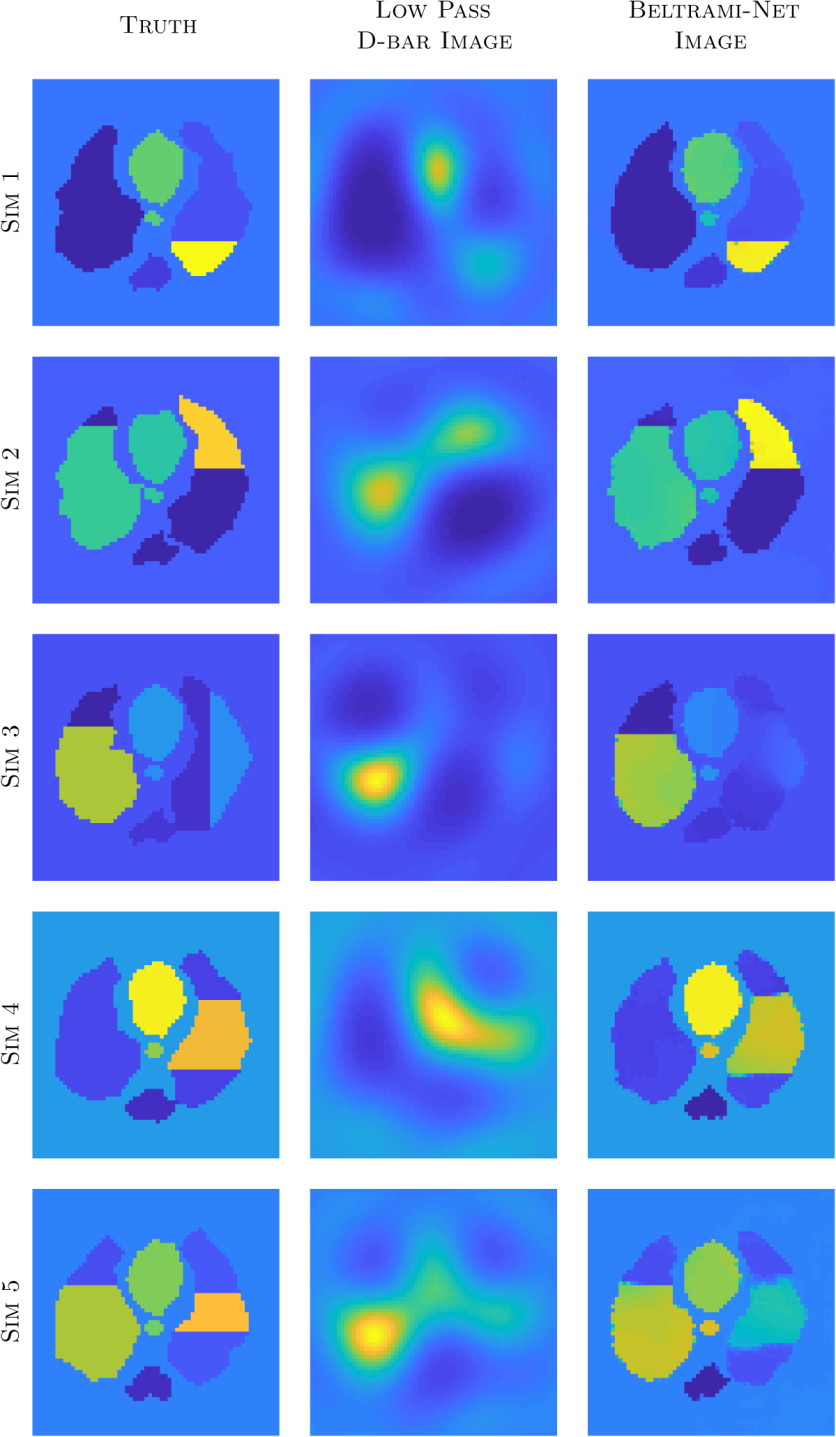
Results for simulated test data with the network trained for the ACT4 data. Note that the training data only included single horizontal divisions in the lungs. Each row is plotted on its own scale.

Figure 6 shows sample low-pass D-bar and Beltrami-net reconstructions from simulated test data for the ACT4 scenario. As it can be seen, if the injuries are consistent with the training, at most a single horizontal dividing line in the lung as in Sims 1–2, the network can almost perfectly recover the targets. If the test data deviates from this convention, Sims 3–5, it is more difficult to recover the correct location and structure, most notably for vertical divisions. Nevertheless, for two dividing lines the network is able locate the conductivity change correctly and establishes a sharp division in the reconstruction.

**Figure 7.**
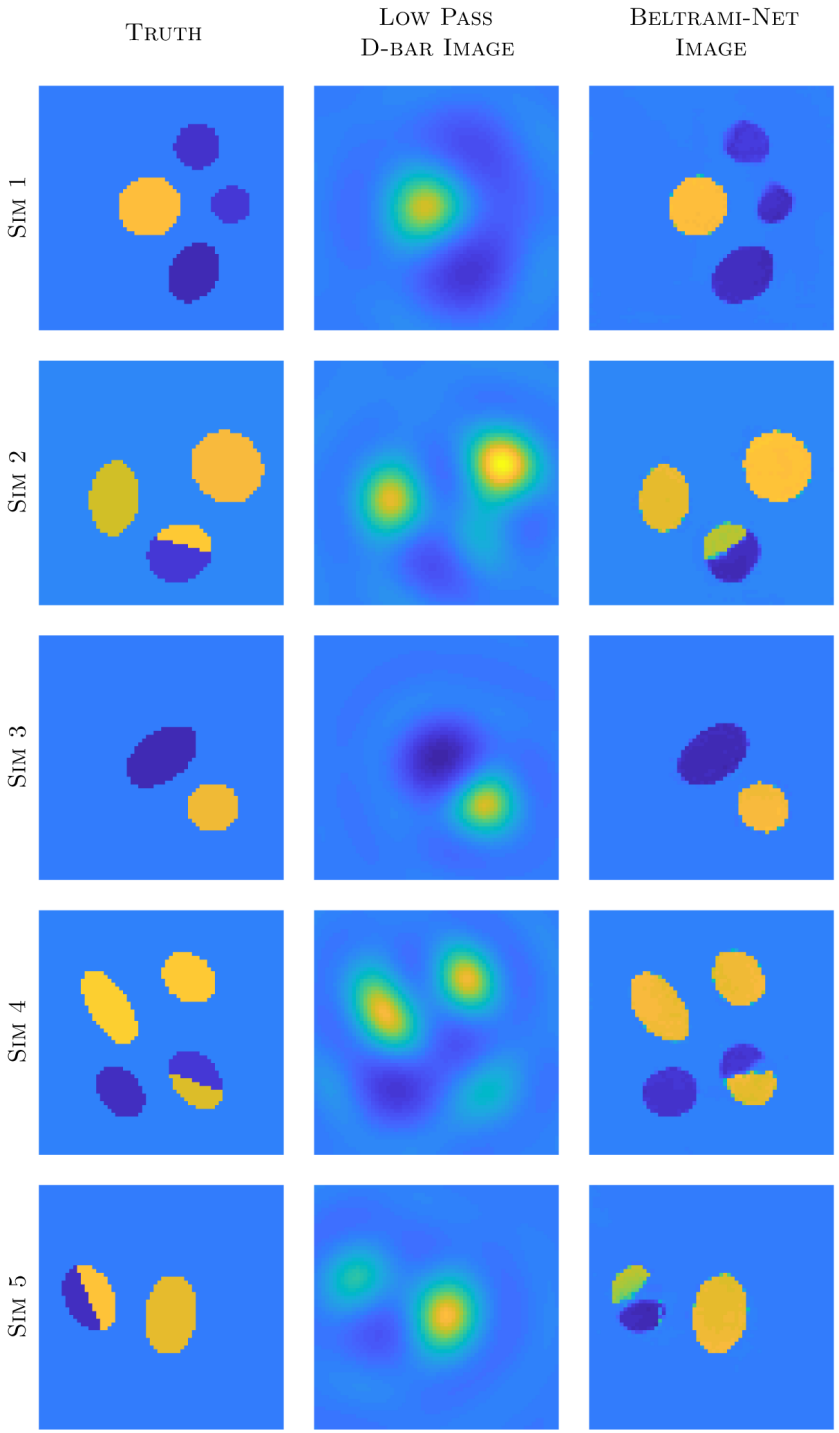
Results for simulated test data with the network trained for KIT4. Note that the training data only included up to three inclusions. All images are on the same scale.

Reconstructions from simulated test data for KIT4 are shown in figure 7. Most notably, if the inclusions are isolated and do not include a cut, the network can reconstruct these very well. We note here that the training data only included up to three inclusions. Nevertheless, the network seems to have no difficulties to reconstruct four inclusions in the image. As can be seen, the cut ellipses are more difficult to reconstruct. In most cases the network manages to include a cut in the ellipse, but in a wrong orientation. In some cases, such as simulation five, the network is not able to distinguish between a cut and two separate inclusions.

### 3.2. Reconstructions from experimental data

We next present reconstructions from the ACT4 and KIT4 experimental data.

#### 3.2.1. Experimental reconstructions from ACT4

**Figure 8.**
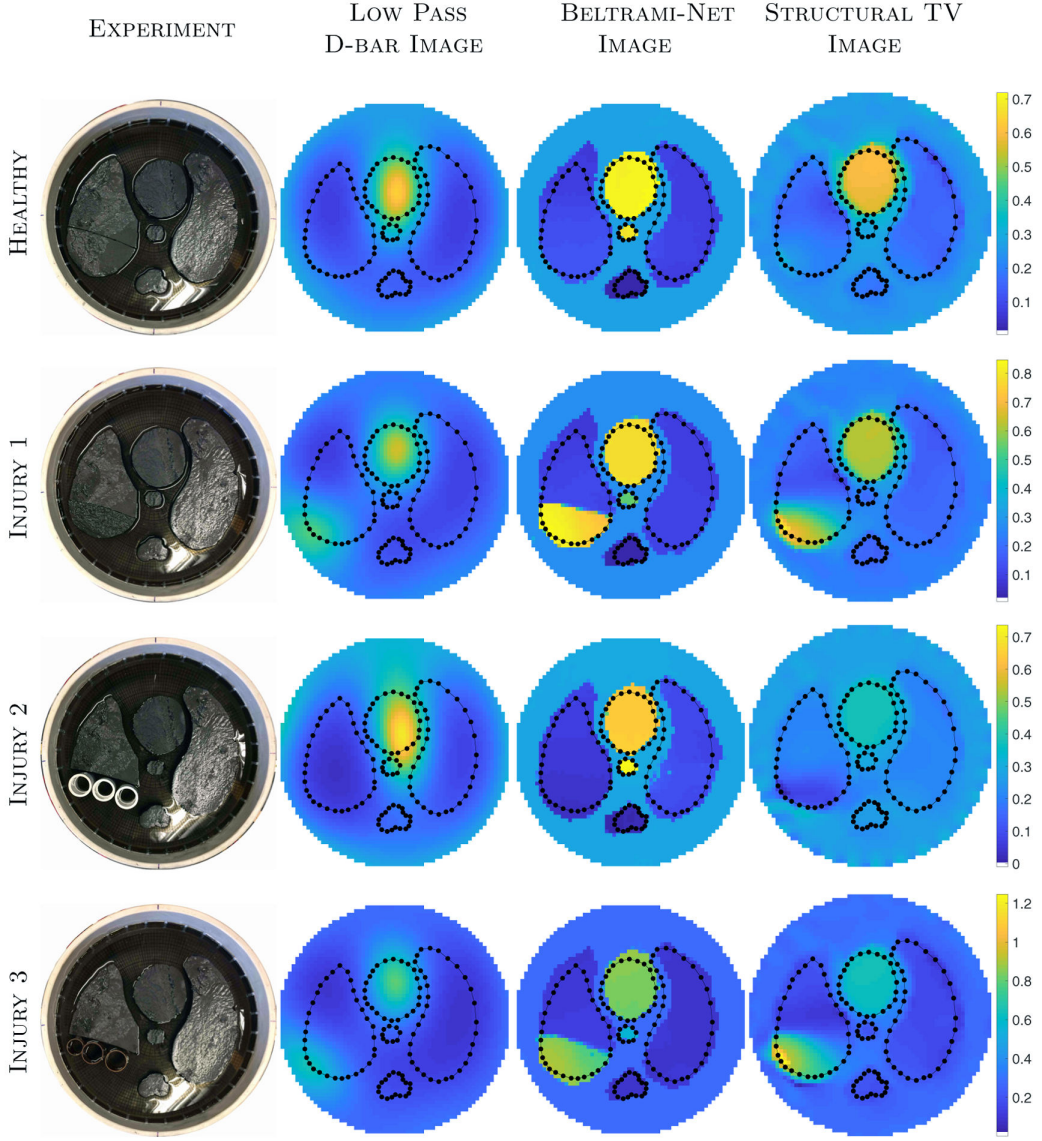
Results for the experimental ACT4 data comparing the initial low-pass D-bar images to the post-processed Beltrami-net images as well as the Structural TV method. Note that Beltrami-net images are displayed here on the circular geometry of the tank, for presentation only. The D-bar images on the full square [−1,1]^2^ were used as inputs to the CNN to produce the Beltrami-net images. The structural TV images did use knowledge of the circular domain shape. Each row is plotted on its own scale.

Figure 8 depicts the results of the *Beltrami-net* approach on four experiments with ACT4 data: Healthy and Injuries 1–3 as shown in figure 1. The black dots represent the approximate boundaries of the ‘healthy’ organs, extracted from the photograph. SSIMs, as well as relative *ℓ*_1_ and *ℓ*_2_ errors, were computed for the experimental reconstructions with the exception of Injury 3, which has infinite conductors (copper tubes). The comparisons, in table 2, used approximate ‘truth’ images formed by assigning the measured conductivity values (table 1) in the respective regions. Note that the coordinates for the bottom portion of the right (DICOM) lung were not specific to each injury, instead the entire region was assigned the same conductivity, even when the injury did not fill up the space as in Injury 2, plastic tubes and Injury 1 which is smaller than the original lung.

The a-EIT reconstructions (10) are computed as references for the Beltrami-net in both, the ACT and KIT4, experiments. In both cases, we aim to construct the matrix field *B*(*p*) such that the amount of prior information would be comparable to the Beltrami-net reconstructions. In case of the ACT experiments, the network is trained using an ensemble of realistic chest images and therefore we chose to use a piecewise constant reference image *p*(*r*) which corresponds to the exact boundary configuration in the healthy case, leading to a situation where (11) is based on more detailed anatomical prior than the Beltrami-net and is labeled ‘Structural TV’ on figure 8.

The obtained reconstructions for the ACT4 scenario are overall of high quality. Visually, we can identify the injuries in the lungs clearly from the Belrami-Net reconstructions as shown in figure 8. Both high conductive injuries are very clearly reconstructed and are even clearly visible in the D-Bar reconstructions and the STV images. The lower conductive injury is harder to identify, in the D-bar reconstruction this results in a overall lower conductivity in the right (DICOM) lung. The Beltrami-net then manages to shift the lower conductivity to bottom of the lung, but cannot establish a sharp boundary. The structural TV image does manage to identify that something of quite low conductivity is occurring in the lower portion of the right (DICOM) lung, however the overall contrast of the image suffers significantly with the heart and aorta reconstructed at values much lower than the truth. We note here, that the Beltrami network was only trained on horizontal injuries, nevertheless it manages to reproduce diagonal cuts for the high conductive injuries. Additionally, the STV reconstructions did not assume injuries in the lungs yet managed to reconstruct them.

Quantitatively, the Beltrami-net reconstructions show clear improvements over the low-pass D-bar reconstructions by all metrics in table 2. We remind here, that this is a case with strong *a priori* knowledge and hence the results are expected to be of very high quality. However, unlike the previous study, Hamilton and Hauptmann (2018), the Beltrami-net method did recover sharp diagonal divisions even when only training on horizontal cuts. The STV reconstructions offered slight to moderate improvements in SSIM, *ℓ*_1_ and *ℓ*_2_ errors over the low-pass D-bar reconstructions for the ‘Healthy’ and ‘Agar’ phantoms. The results for the ‘Plastic’ case were mixed. Overall, the Beltrami-net reconstructions obtained the best SSIMs and lowest *ℓ*_1_ and *ℓ*_2_ errors.

#### 3.2.2. Experimental reconstructions from KIT4

**Figure 9.**
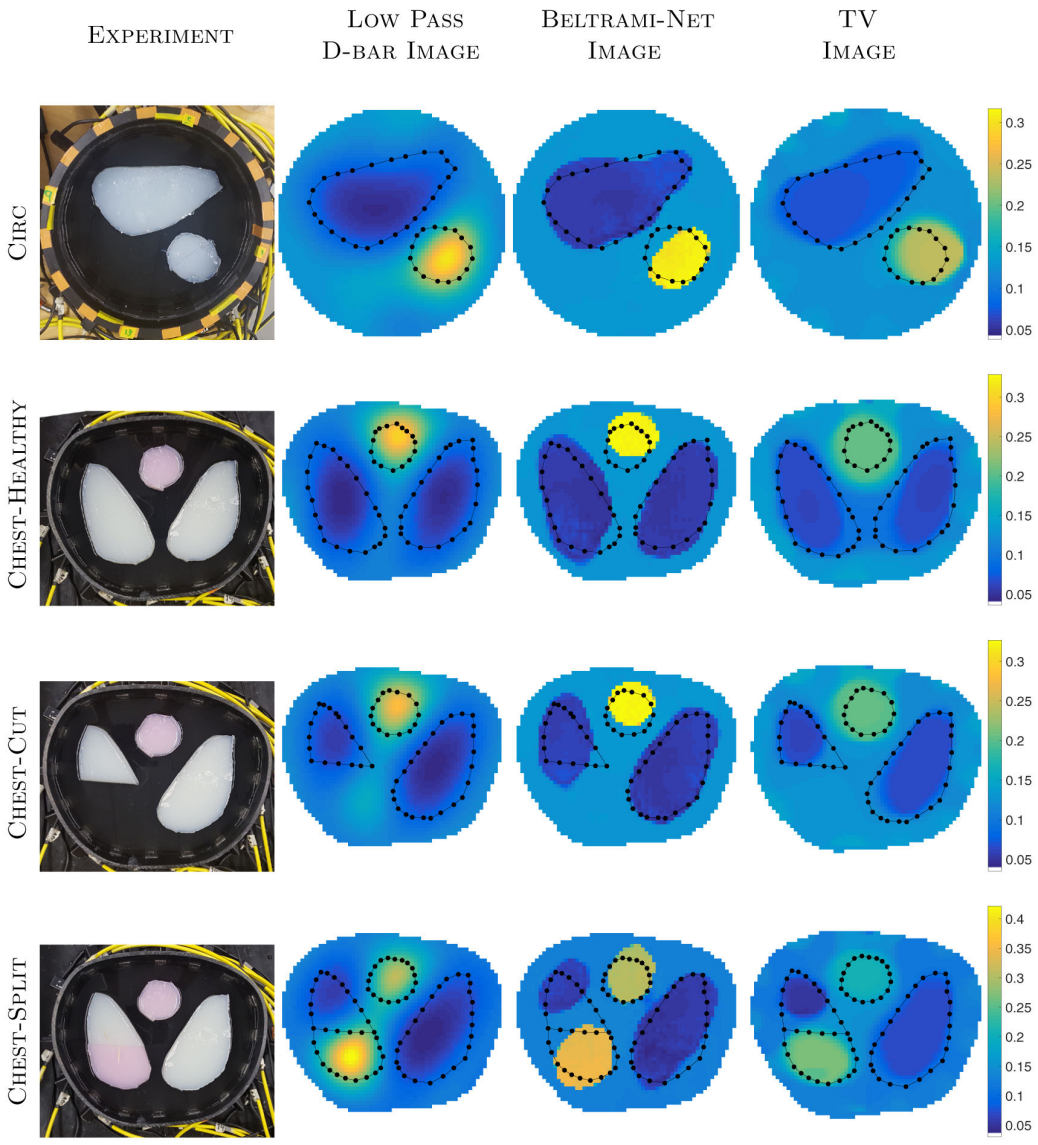
KIT4 Results for the various test scenarios. The initial D-bar image is compared to the Beltrami-net image. The D-bar images, on the full square [−1,1]^2^ are used as the ‘input’ images for the CNN. Images are displayed here clipped to their respective tank geometries for presentation only. Each row is plotted on its own scale.

We next applied the *Beltrami-net* method to the KIT4 datasets corresponding to figure 2 and compared to total variation regularized reconstructions (TV) as outlined in section 2.3. The reconstructed images are shown in figure 9 and quantitative measurements (SSIM and relative *ℓ*_1_ and *ℓ*_2_ images errors) presented in table 3. Note that in the case of the KIT4 data, the network was trained using generic piecewise regular conductivities without prior knowledge about the locations of the edges. For these cases we selected a constant reference image *p*(*r*) = 1 in structured TV regularization (11), leading to *B*(*p*) = *I* and the regularization functional (11) becomes conventional isotropic TV regularization. For clarity, we call such reconstruction ‘TV’ reconstructions for the KIT4 data.

As one can see in figure 9, all three methods produce images where the inclusions are clearly visible. The low-pass D-bar reconstructions are quite blurry as expected, but the post-processed images with Beltrami-net are of very high contrast with sharp edges. In the TV reconstructions, the boundary edges tend to be slightly blurred and there is a clear loss of contrast, which is a quite usual side-effect for TV regularized reconstructions. Neither of the methods is able to identify the split chest in the fourth phantom, and instead separate the lung into the two areas of opposing conductivity with saline between them. We note here that the Beltrami-net was trained with generic prior knowledge of only elliptic inclusions. Nevertheless, the Beltrami-net reconstructions show shapes that differ from this simple prior. Hence we hypothesize that the network mainly learns a segmentation and correction of the existing features in the D-bar reconstructions.

The quantitative measures, SSIM, as well as relative *ℓ*_1_ and *ℓ*_2_ image errors, were computed for each case by comparing to approximate ‘truth’ images constructed using the measured conductivity values and photographs of the experiments, see table 3. The quantitative improvements of Beltrami-net are rather minor in this case. This is as expected due to low prior information. SSIM of D-Bar and Beltrami-net are quite comparable, but generally high already. Most notably, even though the *ℓ*^2^-error is quite constant as well, there is a clear improvement in *ℓ*^1^-error, most likely due to sharper boundary edges. The TV-LS method provides comparable metrics and reconstructions, outperforming both the low-pass D-bar and Beltrami-net methods for the SSIM of the *chest healthy* and *chest cut* phantoms, but underperforming for the *chest split* experiment. Most notably, the Beltraminet reconstruction are consistently better in *ℓ*^1^-error for all provided measures.

### 3.3. Discussion and generalization

The major concern on learned methods for image reconstruction is with respect to their stability under noisy measurement data. This concern is addressed in two ways here. First, the low-pass D-bar algorithm used here is a regularization strategy controlled by the cut-off radius in the scattering data **t**(*k*), or **t**^**exp**^(*k*), which means that there exists a continuous dependence of noise in the measurement to reconstruction error as outlined in Knudsen *et al* (2009). To allow for different noise levels, we have created the training data with varying cut-off radii, that way the network can deal with reconstructions from measurements under different noise. To address the robustness of the second part in the reconstruction procedure, namely the trained networks, we performed the following empirical tests to illustrate the behavior.

#### 3.3.1. Examining robustness of the networks

An established way to examine robustness of networks are via adversarial attacks, where one aims to find a minimal perturbation in the input that leads to a maximal perturbation in the output. Motivated by the study in Antun *et al* (2019), we performed such an adversarial attack on the trained Beltrami-net KIT4 network to examine its stability. That is, given the initial D-Bar reconstruction *σ*^**DB**^ we aim to find a minimal perturbation *δσ* that maximizes the distance in the output, such that

(15) $$\underset{\delta \sigma }{\text{max}}{\left\|G_{\Theta }\left(\sigma^{\text{DB}}+\delta \sigma \right)-G_{\Theta }\left(\sigma^{\text{DB}}\right)\right\|}_{2}^{2}-\alpha \|\delta \sigma {\|}_{2}^{2}.$$

**Figure 10.**
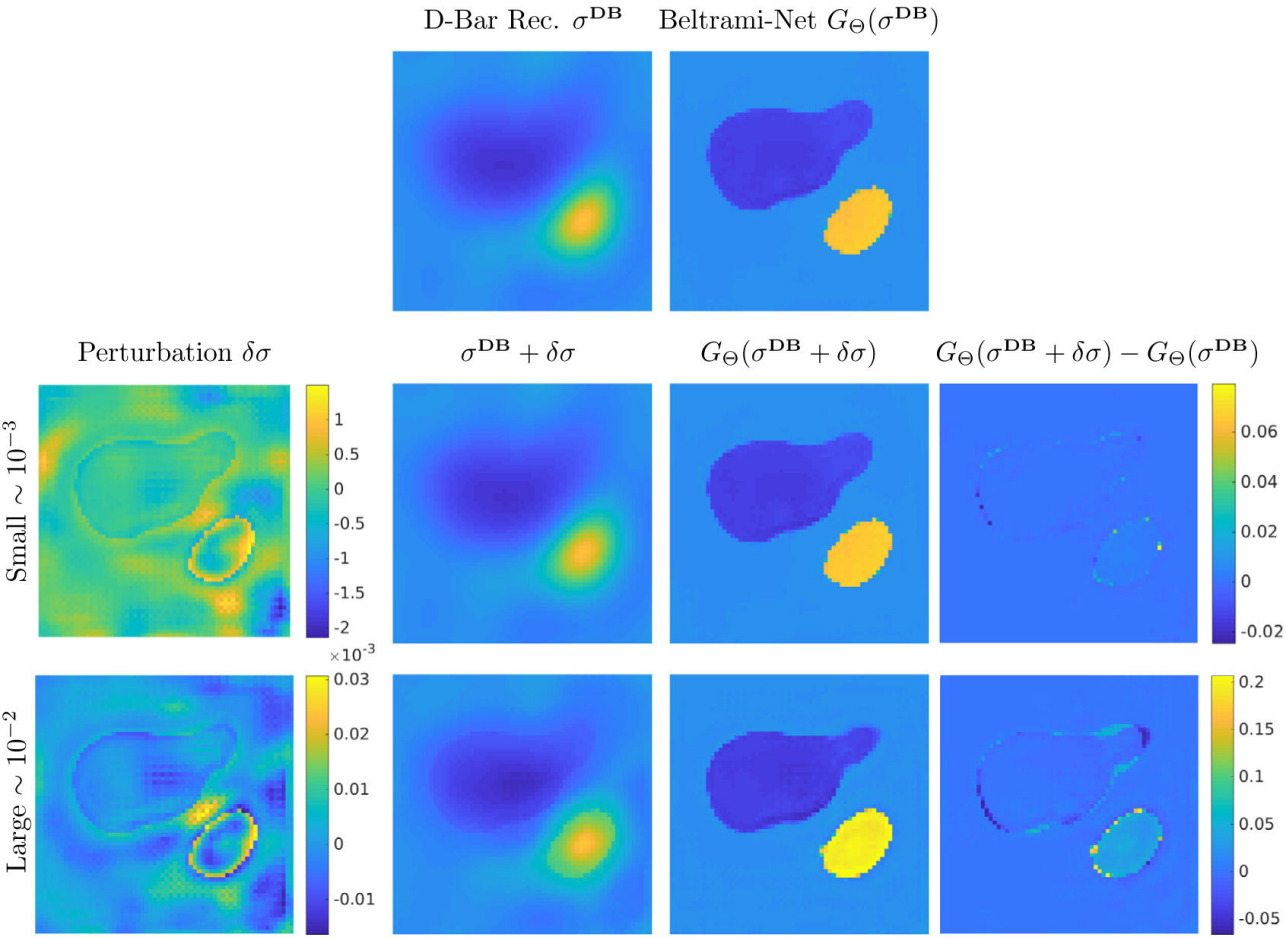
Computation of adversarial perturbations to test network stability, for the KIT4 dataset. The orignial D-Bar reconstruction and Beltrami-net output is shown in the first row. The second row shows a small perturbation, that causes some pixels in the Beltrami-net output to be assigned the background value. The last row shows a very large perturbation, that causes some major parts of the large inclusion to be classified wrongly.

The results for such a test on the KIT4 network are presented in figure 10 for a small and large perturbation found by maximizing (15), where a small perturbation corresponds to an early stage in the maximization of (15) and a large perturbation to a later stage. The perturbations found (left column) led to misclassification in some pixels that would belong to the inclusions, which then led to a large error in the output but to a very small qualitative difference in the image. Even for the large perturbation (bottom row), which in fact produces an input image that is not possible as a low-pass filtered output of the D-Bar reconstruction, the reconstruction by Beltrami-net can be considered qualitatively stable. This illustrates the fact that the network mainly learns a segmentation of the D-bar reconstruction.

**Figure 11.**
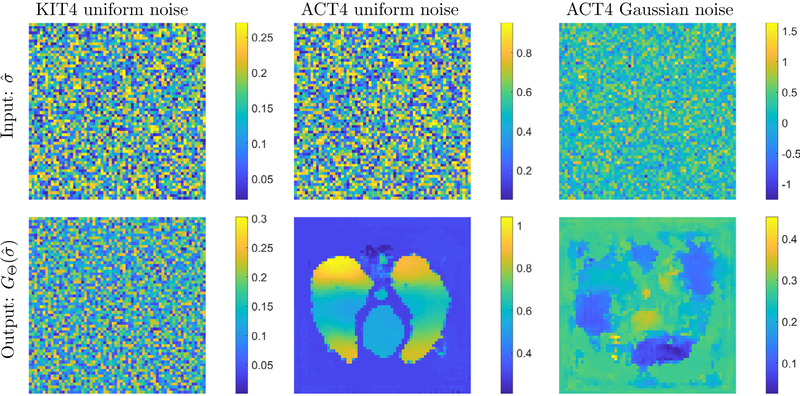
Garbage-in\Garbage-out test on both networks. Random noise (top row) was fed into the two networks and the resulting Beltrami-net results are shown (bottom row). This illustrates the difference in prior information learned by the network. Whereas, the KIT4 network merely learns a segmentation, the ACT4 network learns a specific projection to a thoracic phantom data manifold.

Finally, to illustrate the different nature of the two trained networks, figure 11 presents a ‘Garbage-in\Garbage-out’ test by feeding the network randomly distributed noise. First we tested uniformly distributed noise, such that minimal and maximal values were in the range of the low-pass D-bar reconstructions used to train the respective networks. The result of this experiment, as shown in figure 11, nicely illustrates the different nature of the two networks. The KIT4 network with minimal prior knowledge, i.e. trained only on ellipses therefore only learning a segmentation of the input images, reconstructs ‘garbage’ with the random noise. Whereas the network trained for the ACT4 thoracic reconstructions with strong prior information stands in strong contrast. That network in fact learned a projection of the input images to the data manifold of thoracic phantoms. Thus, the random noise that was in the range of the learned input values was projected onto the data manifold of thoracic phantoms. However, it produced a completely implausible image that can be easily ruled out as an error. On the other hand, if the noise is not in the range, i.e. we chose random Gaussian noise with negative values as shown in column 3, the projection onto the data manifold is not successful and produces a highly corrupted image which can also be ruled out.

#### 3.3.2. Extensions

Whereas the presented approach utilizes the D-bar methodology, specifically without the need of boundary shapes in the training data, the framework can be extended to other reconstruction algorithms. For example, results of non-linear optimization or even linearization-based reconstruction like a single step Gauss–Newton could be used as inputs of learning. In order to retain boundary insensitivity, we suspect that the training data needs to be created with varying boundary shapes.

## 4. Conclusions

In this work we introduced a novel image reconstruction method for absolute EIT that pairs a convolutional neural network with a real-time D-bar method. The training data was computed using the Beltrami equation instead of directly solving the conductivity equation (2) to allow for robustness to changes and uncertainty in domain boundary shape. To demonstrate feasibility, we considered two conceptually different settings: (i) a constrained case of thoracic imaging with the ACT4 measurements, where high *a priori* knowledge is available, and (ii) a very general setting with the KIT4 experiments on varying tank boundary and inclusion shapes with minimal prior knowledge in the training data. Consequently, the obtained results are slightly different in their nature. Whereas the ACT4 reconstructions are of very high quality and close to the target/image prior, the KIT4 reconstructions are more general and it is harder to obtain the exact shapes of the targets, in particular for the ‘chest-cut’ and ‘chest-split’ examples where the sharp divisions in the right (DICOM) lung are smoothed into ellipses. Compared the the reference method of total variation constrained least square reconstructions, the reconstruction quality of Beltrami-net is quite similar with a slight advantage in contrast and hence *ℓ*^1^-error measures.

We believe that this comparison provides good insight of what is possible in EIT in combination with deep learning-based post-processing, in particular for D-bar-based methods. We remind here, that EIT is a highly ill-posed inverse problem and hence it is not surprising that strong prior knowledge is needed to obtain high-quality images. Thus, we believe that the presented approach will be most useful in constrained imaging settings, where boundary shapes might vary, such as thoracic imaging for the identification of lung volumes or injuries. Additionally, process monitoring and non-destructive testing, where knowledge of possible composition and defects is known, may be areas of interest for this approach.

## Figures and Tables

**Table 1.** Conductivity values for ACT4 targets at 3.3 kHz.

	Measured values (S m^−1^)	Simulated values ranges (S m^−1^)
Heart/aorta	0.67781	[0.5, 0.8]
Lungs/spine	0.056714	[0.01, 0.2]
Saline background	0.3	[0.29, 0.31]
Injury 1: agar/graphite	0.67781	[0.01, 1.5]
Injury 2: plastic tubes	0	[0.01, 1.5]
Injury 3: copper tubes	Infinite	[0.01, 1.5]

**Table 2.** Quantitative results for ACT4 experiments: structural SIMilarity indices, as well as relative *ℓ*_1_ and *ℓ*_2_ images errors.

	Low pass D-bar			Beltrami-net			Structured TV		
Experiment	SSIM	*ℓ*_1_-error (%)	*ℓ*_2_-error (%)	SSIM	*ℓ*_1_-error (%)	*ℓ*_2_-error (%)	SIM	*ℓ*_1_-error (%)	*ℓ*_2_-error (%)
Healthy	0.5680	31.43	22.03	0.7296	23.75	13.75	0.6548	30.38	21.27
Agar	0.5176	35.87	24.62	0.6963	27.79	21.01	0.6332	32.56	19.37
Plastic	0.5085	34.91	24.44	0.7053	22.26	13.29	0.5952	37.61	30.08

**Table 3.** Quantitative results for KIT4 experiments.

	Low pass D-Bar			Beltrami-net			TV		
Experiment	SSIM	*ℓ*_1_-error (%)	*ℓ*_2_-error (%)	SSIM	*ℓ*_1_-error (%)	*ℓ*_2_-error (%)	SSIM	*ℓ*_1_-error (%)	*ℓ*_2_-error (%)
Circ agar	0.8831	23.08	14.39	0.8921	19.53	13.11	0.8843	22.09	16.14
Chest healthy	0.8507	26.29	15.73	0.8370	21.03	17.33	0.8709	24.30	17.03
Chest cut	0.8684	22.56	15.55	0.8516	18.67	15.26	0.8939	20.80	16.11
Chest split	0.8244	28.79	14.76	0.8267	21.78	16.90	0.7877	36.28	36.25

## Footnotes

6 Codes will be published on github: https://github.com/asHauptmann/BeltramiNet.

7 www.impedimed.com/products/sfb7-for-body-composition/.
